# The adaptive evolution of virulence: a review of theoretical predictions and empirical tests

**DOI:** 10.1017/S003118201500092X

**Published:** 2015-08-25

**Authors:** CLAYTON E. CRESSLER, DAVID V. McLEOD, CARLY ROZINS, JOSÉE VAN DEN HOOGEN, TROY DAY

**Affiliations:** 1 Department of Mathematics & Statistics, Queen's University, Kingston, ON K7L 3N6, Canada; 2 Department of Biology, Queen's University, Kingston, ON K7L 3N6, Canada; 3 Fogarty International Center, National Institutes of Health, Bethesda, Maryland 20892, USA

**Keywords:** Tradeoff hypothesis, evolutionary medicine, infectious disease

## Abstract

Why is it that some parasites cause high levels of host damage (i.e. virulence) whereas others are relatively benign? There are now numerous reviews of virulence evolution in the literature but it is nevertheless still difficult to find a comprehensive treatment of the theory and data on the subject that is easily accessible to non-specialists. Here we attempt to do so by distilling the vast theoretical literature on the topic into a set of relatively few robust predictions. We then provide a comprehensive assessment of the available empirical literature that tests these predictions. Our results show that there have been some notable successes in integrating theory and data but also that theory and empiricism in this field do not ‘speak’ to each other very well. We offer a few suggestions for how the connection between the two might be improved.

## INTRODUCTION

The virulence of parasites is shaped by evolutionary trade-offs at different biological scales. At the host population scale, parasites that are able to best monopolize susceptible individuals tend to be most successful. However, the ability of a parasite to monopolize susceptible hosts is intimately tied to its ability to persist within hosts and to spread effectively between them. Therefore success at the host population scale is also tied to success at the scale of physiological interactions within a host (van Baalen and Sabelis, 1995*a*; Alizon, 2008*b*; Schmid-Hempel, 2011).

At the same time, a parasite's success at the within-host scale is also shaped by interactions with other, co-infecting, parasite strains and species. These interactions may be antagonistic, when parasites compete for resources or provoke a cross-reactive immune response, or they may be facilitative, when closely related strains cooperate or cotransmit (Pedersen and Fenton, 2007; Lion, 2013; Alizon, 2013*b*). Because parasites must ultimately be successful at both the within- and between-host scales, virulence is expected to evolve to balance these potentially conflicting selection pressures (Mideo *et al.* 2008).

There is now a vast theoretical literature that explores virulence evolution and makes predictions about how we expect virulence to evolve under different conditions. Indeed there are now so many permutations and extensions of mathematical models that it has become difficult to see the forest for the trees. While it would be impossible to comprehensively review all of this theory, we endeavor to highlight some of the key conceptual questions that have been tackled using theory. As we note throughout, comprehensive reviews of the theoretical literature have been written for each of these questions. However, we feel that there is much to be gained from distilling this theoretical literature, in particular, to identify broad-scale, robust, predictions.

Likewise there is an ever-growing empirical literature that aims to test the predictions of theory. Some of these tests are tied very directly to mathematical models, but more often the connections to theory are loose. To highlight the successes and shortcomings of the match between previous theoretical and empirical research, we therefore also discuss the available evidence for each of the broad-scale theoretical predictions that we review.

The structure of our review roughly mirrors the two biological scales of the problem. We begin by considering theory based on trade-offs at the between-host scale. We focus primarily on the trade-off between parasite virulence and transmission, reflecting the theoretical attention paid here (Alizon *et al.* 2009). In this section, we ignore any theory that includes either multiple infections (e.g. Gandon *et al.* 2001*a*) or an explicit dynamical consideration of within-host processes (e.g. André *et al.* 2003). We then consider how multiple infections influence the evolution of virulence, highlighting especially how and why multiple infection can alter predictions based on trade-off theory. Specifically, we review models of superinfection, coinfection and kin selection.

Each section of the review is relatively self-contained to provide readers with bite-sized chunks of the literature to digest. As will be seen, one of the take-away messages of this review is that, to a large extent, theory and empiricism in this field do not ‘speak’ to each other very well. Experiments seldom measure the traits explored by the theory, and theory seldom models the traits measured by empiricists. Although neither empiricists nor theoreticians are particularly to blame for this miscommunication, what is sorely needed at this point is a tighter integration of mathematical modeling with empirical research. We close our review by highlighting recent work in this vein (Mideo *et al.* 2011; Berngruber *et al.* 2013, 2015).

## DEFINING VIRULENCE

Before we begin, it is necessary to define what we mean by virulence. The most general definition of virulence is the reduction in host fitness caused by infection (Read, 1994). This definition, ironically, may be a primary reason for the lack of integration between theory and data in this field: whereas fitness can be quantified precisely in a mathematical model, it is exceedingly difficult to measure empirically (Metcalf *et al.* 2015). In mathematical models, virulence is quantified as an infection-induced increase in host mortality rate or reduction in host reproductive rate. In experimental or observational data, virulence is often quantified by measures of ‘harm’ done by the parasite, such as host anemia, weight loss, or morbidity, assuming that this harm is correlated with negative impacts on host fitness. However, the relationship between these metrics and host mortality is not typically straightforward. Even more troubling, common empirical measures of infection-induced mortality (such as case mortality rate or lethal dose) do not have a simple relationship with the theoretical measure (instantaneous mortality rate); as such, the evolutionary response of these empirical measures can be opposite in direction to that of the theoretical measure (Day, 2002*a*).

Throughout our review of theory, ‘virulence’ will typically refer to the instantaneous mortality rate caused by infection, unless otherwise noted. Although parasites often affect host reproduction, the overwhelming majority of modeling work has studied the evolution of virulence as host mortality rate. This is because, in the absence of spatial structuring, host reproduction does not effect parasite fitness unless a relationship between host reproduction and parasite transmission is assumed (O'Keefe and Antonovics, 2002) or there is coevolution between hosts and parasites (Best *et al.* 2010). For the empirical literature, we will explicitly state how virulence was quantified. As will be seen, the match between the theoretical and empirical definitions of virulence is often only approximate.

## TRADE-OFFS AND THE EVOLUTION OF VIRULENCE

Trade-offs between different components of parasite fitness provide the dominant conceptual framework for understanding the adaptive evolution of virulence (Alizon *et al.* 2009). This idea was first introduced in Anderson and May (1982) to help explain patterns in myxomatosis data, and by Ewald (1983) to explain the severity of vector-transmitted disease. It arises quite naturally from a consideration of the classic expression of parasite fitness, *R*_0_. The *R*_0_ expression for a simple SIR epidemiological model is illustrative:

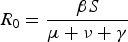


Here, parasite fitness is the product of the rate at which new infections are caused by an infected host (*βS*) and the duration of infection (*μ* + ν + *γ*)^−1^ (Bremmerman and Thieme, 1989). In this formulation, *β* is the transmission rate, *S* is the density of susceptibles, *γ* is the rate at which an infected host clears the disease, *μ* is the background mortality rate and ν is the mortality rate due to infection, often referred to as ‘virulence’. More precisely, *β* is the rate of contact between susceptible and infectious individuals multiplied by the probability of transmission per contact; it has units of individual^−1^ × time^−1^. Parasite fitness is increased by increasing transmission (i.e. increasing the value of *β*) and/or by prolonging the infection (e.g. decreasing mortality ν or clearance *γ*). Trade-off theory assumes that a parasite cannot simultaneously increase transmission and prolong infection, and so parasites are attempting to maximize *R*_0_ subject to these constraints. Although within-host processes may not be explicitly represented in the models, epidemiological trade-offs are thought to emerge from the dynamics of the within-host interactions between the immune system and parasite.

By far, the most widely studied trade-off involves transmission and virulence (Anderson and May, 1982; Frank, 1996; Alizon *et al.* 2009). Transmission and virulence are linked by within-host replication: increasing parasite abundance increases the likelihood of transmission, but also increases the likelihood of host death; mathematically, this assumption can be formalized by making transmission rate *β* an increasing function of parasite-induced mortality rate ν. Nearly all of the literature we summarize below assumes this trade-off. However, another potential trade-off suggested by an examination of *R*_0_ involves virulence and recovery rate (Anderson and May, 1982; Frank, 1996). This trade-off is also mediated by replication rate, with high abundance increasing the likelihood of host death, but also decreasing the likelihood of the host clearing the infection (Antia *et al.* 1994); mathematically, this assumption makes recovery rate *γ* a decreasing function of parasite-induced mortality rate ν.

Despite the importance of these trade-offs to the theory of virulence evolution, empirical demonstrations of trade-offs are surprisingly uncommon. This imbalance between importance and evidence has led some to question the relevance of the trade-off model (Ebert and Bull, 2003; Bull and Lauring, 2014). As noted above, this imbalance may be due to the difficulty of conducting the appropriate experiments, rather than an actual lack of relevance (Alizon *et al.* 2009; Alizon and Michalakis, 2015). Moreover, few of the experiments purporting to test the assumptions of the trade-off model, including those reporting evidence for the trade-offs (see Table 12.4 in Schmid-Hempel, 2011), actually measure the traits involved in the mathematical theory, specifically host mortality rate, transmission rate, or host recovery rate.

However, evidence for trade-off theory should also be sought from experiments and observations that test its predictions. As noted by Frank and Schmid-Hempel (2008), trade-off theory is essentially comparative: predictions for how virulence evolves in response to changes in ecological context (such as host mortality rate) can be tested experimentally or by comparing populations of the same species or of very closely related species (situations where it is reasonable to assume that both are subject to the same trade-off). Here we review some of the well-studied predictions of trade-off theory. We look at the predictions, and experiments testing those predictions, for how virulence evolves in response to transmission mode, host mortality, epidemiological dynamics, spatial structure and vaccination. For each topic, we first lay out the general prediction of trade-off theory, articulate any nuances discovered by mathematical models, and then summarize the experimental evidence for these predictions.

## Transmission mode

One of the earliest predictions of transmission–virulence trade-off theory was that virulence will be higher in parasites with a transmission mode other than direct transmission, such as environmental or vector transmission (Ewald, 1983). The reasoning behind this prediction was straightforward: if a parasite does not rely on direct host–host contact for transmission, it does not pay as severe a fitness cost for increasing host mortality as would a directly transmitted parasite. This verbal model has been formalized into models that make different assumptions about the particulars of transmission, making this one of the best-studied topics in virulence evolution. Although these theoretical studies have uncovered nuances to the general prediction, most of the experimental tests have focused on comparing virulence across populations or experimental treatments that vary the relative contribution of direct and non-direct transmission.

### Environmental transmission

If parasites produce propagules that can survive in the environment for a long time, host mortality should impose a weaker constraint on virulence evolution (Ewald, 1983); by extension, the longer the parasites can persist in the environment, the more virulent they can evolve to become. This hypothesis has become known as the ‘Curse of the Pharoah’, in reference to the mysterious death of Lord Carnavon after entering the tomb of the pharaoh Tutankhamen (Bonhoeffer *et al.* 1996). While intuitive, initial mathematical formalizations of this hypothesis, which considered the evolution of parasites that transmit only through the environment, found that this simple prediction only holds when the system is not in equilibrium (Bonhoeffer *et al.* 1996). This was later explained as a consequence of the fact that propagule longevity in the environment primarily affects the rate of infection, which only matters for parasite evolution during epidemics (Frank, 1996; Gandon, 1998). Later theory, however, has elaborated on these simple models and found several conditions under which the ‘Curse of the Pharoah’ hypothesis is valid, and increasing propagule longevity increases virulence. These include if parasites transmit both directly and indirectly through the environment (Day, 2002*c*); if parasites transmit only after host death (Day, 2002*c*; Day and Gandon, 2006); or if multiple infection is possible (Gandon, 1998; Day and Gandon, 2006). The consensus of mathematical theory based around a transmission–virulence trade-off, therefore, is that increasing environmental transmission will lead to the evolution of increased virulence under most scenarios. Models considering other trade-offs have come to a similar conclusion, such as trade-offs between virulence and survival in the environment (Caraco and Wang, 2008) or between growth in the host and growth in the environment (Brown *et al.* 2012).

Empirical evidence for the Curse of the Pharoah hypothesis comes primarily from comparative studies. For example, data from waterborne parasites like *Vibrio cholerae* and *Salmonella* suggests a positive correlation between the case mortality and the fraction of cases caused by environmental transmission (Ewald, 1991). An analysis of *V. cholerae* strains suggests that strains recovered from locations (or times) with better water sanitation have lower virulence, perhaps because improving water sanitation reduces the opportunity for environmental transmission, thereby increasing the importance of direct transmission (Ewald, 1994). A meta-analysis of 16 human respiratory tract parasites (Walther and Ewald, 2004) found a positive correlation between environmental persistence and case mortality, with highly virulent parasites like smallpox virus and *Mycobacterium tuberculosis* having much longer environmental persistence times than avirulent parasites like rhinovirus and *Haemophilius influenzae*. On the other hand, a comparison of 16 different phages of *Escherichia coli* found that virulent phages (virulence was defined as the burst size divided by the time to lysis) had reduced environmental persistence (higher rates of inactivation), in apparent contradiction to the general theoretical expectation that more virulent parasites would persist longer in the environment (De Paepe and Taddei, 2006). Of course, the typical caveat about correlation and causation applies here; as suggestive as these correlational studies are, they do not control for other factors that can influence parasite virulence evolution, such as the potential for superinfection (Bonhoeffer *et al.* 1996). Experimental evidence against the prediction was also found in a study of vesicular stomatitis virus (Ogbunugafor *et al.* 2013). In that study, the authors allowed the virus to evolve in different host cell lines; virus evolved in carcinoma cells was better able to persist outside of host cells but had reduced virulence (measured by plaque size), whereas virus evolved in non-cancer cells had reduced environmental persistence and increased virulence. This suggests the existence of a trade-off between persistence and virulence, a trade-off which is not included in previous theory (e.g. Caraco and Wang (2008) assume that virulence is positively correlated with environmental persistence). However, the strongest evidence, for or against, would come from experimental studies that directly manipulate transmission mode, but we are unaware of any such studies.

Note that all theoretical work summarized here assumes parasites are obligate rather than opportunistic. When parasites can grow in both the environment and the host (i.e. opportunistic parasites), none of these conclusions would necessarily hold. The addition of growth in the environment effectively creates another selective arena; thus how virulence should evolve will critically depend upon whether or not there is any linkage between virulence within-host and survival or growth in the environment. For example, empirical work on opportunistic parasites shows that selection for anti-predator adaptations in the environment can lead to decreased within-host virulence (Mikonranta *et al.* 2012), whereas in a different system, starvation in the environment caused higher within-host virulence (Sundberg *et al.* 2014). Indeed, growth in the environment would possibly foster evolutionary diversification of the parasite into strains specializing in the different niches (i.e. within-host *vs* environment).

### Vector transmission

Following a similar line of reasoning, verbal models developed primarily by Ewald predicted that vectored parasites should also have higher virulence than directly transmitted parasites, and that virulence should be lower in the vector than the main host (Ewald, 1983, 1994). The rationale for these predictions is straightforward: increased mortality of the main host need not reduce transmission because the vector causes transmission between main hosts, and the parasite should use the main host for ‘amplification’ and the vector for ‘dispersal’ (Ewald, 1994, p. 47). However, theories formalizing these intuitive hypotheses have shown that neither is valid in general (van Baalen and Sabelis, 1995*b*; Day, 2001, 2002*b*; Elliot *et al.* 2003). Although the processes put forward in Ewald's verbal argument play a role, direct and vector transmission also differ in how the transmission route changes throughout an infection as a host becomes ill; how virulence will evolve depends on the way that virulence affects such temporal changes in transmission.

Empirical evidence for these predictions is equivocal. Correlational evidence for the hypotheses has been found for some human diseases (Ewald, 1983, 1994). A meta-analysis of studies of vector-borne parasites of plants have found little evidence that these systems follow the transmission–virulence trade-off, in which case the predictions of the models may not be relevant (Froissart *et al.* 2010). And while there is clear evidence that parasites often have virulent effects on their vectors (Ferguson and Read, 2002; Lambrechts and Scott, 2009), no studies have compared the relative virulence of parasite to its host *vs* its vector. Additionally, there is the potential for conflicting selection pressures to shape virulence evolution, as was the case for environmentally transmitted parasites. In particular, because the parasite inhabits more than one environment, it is possible for adaptation to one environment (e.g. the vector) to influence virulence expression in the other (e.g. the host). Thus, on the basis of both theory and data, it is unclear that any simple predictions will hold for vector-borne diseases.

### Vertical transmission

The simple prediction for parasites that can be transmitted from parent to offspring is that vertical transmission should select for reduced virulence: because the parasite depends on host survival and reproduction for its transmission, virulence that increases mortality or reduces fecundity will be under strong negative selection (Ebert, 2013). In reality, however, almost all parasites that are transmitted vertically can also be transmitted horizontally.

In fact, theory predicts that strict vertical transmission should be exceedingly rare because of the extinction risk posed to parasites relying on purely vertical transmission (Lipsitch *et al.* 1995*b*; Altizer and Augustine, 1997) (although this extinction risk can be mitigated if being infected with a vertically transmitted parasite ‘vaccinates’ a host against infection by a different, horizontally transmitted parasite; Berngruber *et al.* (2013); Haine (2008); Jones *et al.* (2007); Jones *et al.* (2011); Lipsitch *et al.* (1996)). Mixed mode transmission is predicted to select for an evolutionary reduction in virulence, regardless of which transmission pathway is more common (Sasaki and Iwasa, 1991; Lipsitch *et al.* 1995*b*, 1996; Frank, 1996; Day and Proulx, 2004). This is because of an epidemiological feedback: regardless of which transmission pathway is more common, increasing transmission will increase infection prevalence. As prevalence increases, the relative importance of vertical transmission also increases (in particular, at a prevalence of 100%, all transmission is vertical assuming multiple infection is not possible). This causes an evolutionary decrease in virulence. In general, the expectation is that horizontal transmission (and higher virulence) will evolve when susceptible hosts are common, whereas vertical transmission (and lower virulence) will evolve when susceptible hosts are uncommon, especially if host fecundity is high (Lipsitch *et al.* 1996; Berngruber *et al.* 2013, 2015).

Evidence from both correlational and experimental studies strongly support these predictions. A comparative study of nematode parasites of fig wasps showed a positive correlation between virulence (measured as the reduction in host lifetime reproductive success) and horizontal transmission (Herre, 1993). Studies manipulating the relative amount of vertical *vs* horizontal transmission have shown that increasing vertical transmission reduces virulence, whereas increasing horizontal transmission increases virulence (Bull *et al.* 1991; Agnew and Koella, 1997; Messenger *et al.* 1999; Stewart *et al.* 2005; Pagán *et al.* 2014). Similarly, a study manipulating the spatial structure of the host population showed, both theoretically and experimentally, that when the host population was well-mixed, horizontally transmitting, highly virulent parasites dominated; when the host population was structured, susceptible host density was lower, and vertically transmitted, low virulence parasites dominated (Berngruber *et al.* 2015). Experimental evolution studies have shown that, as predicted by the theory, vertical transmission and low virulence evolve in environments with high host fecundity (Magalon *et al.* 2010; Dusi *et al.* 2015); indeed, empirical evidence suggests that some parasites can plastically switch between vertical- and horizontal-transmission, favoring vertical transmission in rapidly replicating hosts and horizontal transmission when host population growth is low or negative (Agnew and Koella, 1999; Kaltz and Koella, 2003). Studies have also found trade-offs between transmission pathways, such that being good at horizontal transmission reduces the ability to transmit effectively vertically, and vice versa, with attendant changes in virulence (Turner *et al.* 1998; Stewart *et al.* 2005). These trade-offs between transmission modes may tip the evolution of symbiotic organisms towards either avirulence and primarily vertical transmission, or virulence and primarily horizontal transmission (Ebert, 2013).

## HOST MORTALITY

Classic life history theory predicts that reducing lifespan will increase reproductive effort at earlier ages (Gadgil and Bossert, 1970; Charlesworth, 1994). Analogously, because host death is typically death for the parasite (except for obligate killers), increased host mortality should select for increased transmission (the parasite equivalent of birth); assuming a transmission–virulence trade-off that would mean an evolutionary increase in virulence. Indeed, this is one of the most widely accepted predictions of trade-off theory. Early theoretical work making this prediction focused on host background mortality rate (Anderson and May, 1982; Kakehashi and Yoshinaga, 1992; Lenski and May, 1994; Ebert and Weisser, 1997; Gandon *et al.* 2001*a*). However, the prediction was also found to hold if increased mortality was caused by interaction with a predator (Morozov and Adamson, 2011) or the host's immune response (Day *et al.* 2007), unless virulence directly influenced these mortality rates. Generally, if parasite virulence influences host mortality from sources other than parasitism, more complicated responses are possible (Williams and Day, 2001). For example, if increased parasite virulence increases predation risk, then the evolution of decreased virulence (Choo *et al.* 2003), evolutionary branching of virulence (Morozov and Best, 2012), and evolutionary cycles of virulence (Kisdi *et al.* 2013) are all possible. Alternatively, if parasite virulence increases immune self-harm (i.e. immunopathology; Long and Graham, 2011), then increasing the mortality cost of immunopathology can select for an evolutionary decrease in virulence (Day *et al.* 2007). A fairly tight set of predictions emerges from this theory: unless virulence influences mortality from other sources, increasing host mortality will select for increased virulence.

Despite the large number of theoretical studies on this question, and the relatively straightforward experimental design they suggest to test their predictions, there have been surprisingly few empirical demonstrations of these predictions. Experimental evolution studies show evidence for both increased and reduced virulence in response to increased host mortality. Serial passage experiments that experimentally manipulated host life span have shown that parasites under selection imposed by early host mortality evolve to be more virulent (inducing higher mortality) than parasites under selection imposed by late host mortality (Cooper *et al.* 2002; Nidelet *et al.* 2009; Wasik *et al.* 2015). On the other hand, Ebert and Mangin (1997) found that replacing 80% of the host population, a slightly unusual form of mortality, caused an evolutionary decrease in virulence. This result was later explained as a consequence of high mortality reducing within-host competition between strains (Gandon *et al.* 2001*a*). Increased mortality due to culling has also been implicated in the increase in virulence seen in avian influenza circulating in ducks (Chen *et al.* 2004; Shim and Galvani, 2009). In fact, given that culling is a common practice in agricultural systems and that these systems are important sources of zoonotic disease, more empirical work investigating the role of mortality in shaping virulence evolution is warranted (Mennerat *et al.* 2010).

## EPIDEMIOLOGICAL DYNAMICS

The primary theoretical framework for studying virulence evolution focuses on long-term predictions, essentially assuming a separation of timescales between epidemiological and evolutionary dynamics. Given the fact that evolution is likely to be quite fast for many parasites, with short generation times, large population sizes, and (for viruses especially) high mutation rates, this assumption is likely overly restrictive. Moreover, this framework necessarily ignores potential evolutionary changes in virulence that might occur when epidemiology and evolution happen at similar rates. However, a number of authors have studied how virulence evolves during the epidemic, rather than endemic, phase of epidemiological dynamics (Lenski and May, 1994; Day and Proulx, 2004; Day and Gandon, 2007; Bull and Ebert, 2008; Bolker *et al.* 2010). The consensus from this theoretical work is that, under a transmission–virulence trade-off, virulence will be higher during the early stages of an epidemic, when the abundance of susceptible hosts is high, and will evolve to lower levels as the endemic equilibrium is reached. Put another way, during an epidemic, the parasite population will be dominated by more virulent parasites than would be expected on the basis of the standard theoretical approach. This has important implications for understanding the dynamics of emerging infectious disease (Berngruber *et al.* 2013).

Experimental or observational evidence for this prediction is challenging to find, as it requires both that virulence is measured over the course of an epidemic and the ability to rule out alternative explanations for changes in virulence, such as host evolution or parasite evolution in response to other factors, like medical intervention. The classic reference for rapid evolution of virulence is of course the rabbit-*myxoma* system, but host evolution is a confounding factor (Fenner and Ratcliffe, 1965). Similarly, HIV virulence is also rapidly evolving, but the primary drivers are not entirely understood (Fraser *et al.* 2014; Payne *et al.* 2014). An empirical example comes from work with the bacteriophage *λ* (Berngruber *et al.* 2013). This phage can either lyse the host cell and transmit horizontally, or integrate itself into the host genome and transmit vertically. Working with two strains of *λ*, one that mostly transmits vertically and one that mostly transmits horizontally, they found that the virulent (horizontally transmitted) phage dominated early in an epidemic, but as prevalence increased, the less virulent (vertically transmitted) strain won out. One of the strengths of this study was that it was paired with a mathematical model for the system that predicted, a priori, both the qualitative and quantitative dynamics of virulence evolution.

## SPATIAL STRUCTURE

The overall consensus from the theoretical literature is that lower virulence will evolve in populations with restricted spatial movement, and higher virulence will evolve in populations with greater connectivity (Claessen and de Roos, 1995; Lipsitch *et al.* 1995*a*; Boots and Sasaki, 1999, 2000; Haraguchi and Sasaki, 2000; Boots *et al.* 2004; Caraco *et al.* 2006; Kamo *et al.* 2007; Messinger and Ostling, 2013); this prediction holds even if virulence affects host reproductive output rather than mortality. The explanation for this prediction is multifaceted (Lion and Boots, 2010). Assuming a positive correlation between virulence and transmission, high virulence parasites will tend to ‘self-shade,’ rapidly depleting the local pool of susceptible hosts until most infected hosts are surrounded by other infected hosts (Boots and Mealor, 2007). If connectivity is low, self-shading guarantees that these clusters of hosts infected with parasites of high virulence will transmit very little. Low virulence parasites, on the other hand, will tend to find themselves surrounded by clusters of susceptible hosts, leading to selection for reduced virulence as susceptible host density increases (Boots and Sasaki, 1999). Moreover, clusters of infected hosts run a high risk of local extinction before finding a new cluster of susceptible hosts to infect, producing a competition-persistence trade-off (Messinger and Ostling, 2009). Additionally, if most interactions are local, it is likely that parasites will interact primarily with close relatives. In this case, lower virulence and transmission can evolve as a consequence of kin selection: even though reduced virulence may carry a direct fitness cost, it is more than compensated for by the inclusive fitness benefits reduced transmission has on relatives competing for the same pool of susceptible hosts (van Baalen, 2002; Wild *et al.* 2009; Lion and Boots, 2010).

These theoretical predictions have considerable experimental support. Several studies in bacteria-phage systems have shown that restricted migration favors less virulent strains (Dennehy *et al.* 2007) and leads to the evolution of low virulence (Kerr *et al.* 2006; Eshelman *et al.* 2010; Berngruber *et al.* 2015). In these experimental evolution studies, self-shading was essentially imposed under the restricted migration protocol, as only parasites on boundaries between infected and uninfected clusters could infect new hosts. This self-shading led to the evolution of reduced virulence compared with unrestricted migration (Eshelman *et al.* 2010). Boots and Mealor (2007) indirectly manipulated transmission by changing host movement; they found that restricted host movement led to the evolution of reduced infectivity (infectivity is a reasonable proxy for virulence in this system, as the main transmission route is through cannibalism of infected corpses and infectivity was defined by the per cent mortality).

## VACCINATION

Literature on the evolutionary consequences of vaccination tends to focus either on the consequences of vaccination for parasite life history evolution (e.g. if we vaccinate using a vaccine that reduces parasite growth, how does parasite virulence evolve? Gandon *et al.* 2001*b*) or the consequences of vaccination for producing ‘escape mutants’ (e.g. if we vaccinate against strain 1, what is the probability that strain 2 will spread? McLean, 1995). Given the scope of this review, we will focus on the literature dealing with the first question, but we point out that these two topics are closely related and can be studied with the same epidemiological evolutionary framework (Gandon and Day, 2007).

The goal of vaccination is, of course, to limit the harm done to the host population. Importantly, however, there are two possible approaches to achieving this goal. The classical approach is to target pathogen fitness, either by preventing infection, limiting parasite within-host growth, or preventing transmission from infected hosts (Gandon *et al.* 2001*b*). More recently, approaches that target the damage done by parasites, without directly targeting the parasite itself, have been explored as alternatives (Vale *et al.* 2014). This distinction is akin to the distinction between resistance and tolerance mechanisms in the eco-immunology literature (Schneider and Ayres, 2008). It is important to understand how vaccines with different modes of action drive virulence evolution. Here we will refer to vaccines as either anti-fitness (if they directly target the parasite) or anti-damage (if they target the harm done by the parasite), and discuss the theoretical and empirical literature exploring how each class of vaccine shapes virulence evolution. A secondary issue that arises in vaccination is whom to vaccinate, if the host population is divided into more and less vulnerable subpopulations, with the more vulnerable population generally experiencing higher mortality, lower clearance and higher rates of infection and transmission. It turns out that this choice also has implications for vaccine-driven virulence evolution, because vaccination (of any type) of one subpopulation will concentrate cases in the unvaccinated subpopulation. This causes parasite virulence to adapt primarily to the characteristics of this subpopulation, which can often reverse the predictions from theory focusing on uniform vaccine coverage (Williams and Day, 2008).

### Antifitness vaccines

As noted above, vaccines can reduce parasite fitness by preventing infection, reducing within-host growth, or preventing transmission. These have been shown to have very different evolutionary consequences for parasite virulence evolution (Gandon *et al.* 2001*b*). Vaccines which reduce within-host growth rates are predicted to increase virulence (Gandon *et al.* 2003, 2001*b*; André and Gandon, 2006; Ganusov and Antia, 2006). In studies using epidemiological models (models focused at the between-host level), virulence increases because reducing parasite growth rate reduces the risk of host death (assuming that parasite-induced mortality depends on parasite replication); reducing the risk of death for infected hosts reduces the cost of virulence, causing an evolutionary increase in virulence (Gandon *et al.* 2003, 2001*b*). Vaccines that either block infection or block transmission, on the other hand, have no effect on virulence evolution unless multiple infection is possible (Gandon *et al.* 2003, 2001*b*; Gandon and Day, 2007).

### Antidamage vaccines

Again, we can distinguish different modes of action for vaccines that reduce the damage done to the host (Vale *et al.* 2014). Some vaccines limit damage by targeting virulence mechanisms, such as toxins and other virulence factors, as in vaccines against diphtheria and pertussis (Soubeyrand and Plotkin, 2002). These vaccines may work by neutralizing these factors or by preventing their expression altogether. Alternatively, vaccines may limit damage by improving host health without targeting any aspect of the parasite.

Vaccines that reduce damage, either by reducing toxin production or targeting host health, will lead to the evolution of high-virulence parasites because they reduce the cost of virulence to the parasite (Gandon *et al.* 2001*b*; Atkins *et al.* 2013; Vale *et al.* 2014). On the other hand, if vaccines simply neutralize virulence factors, the parasites still bear the cost of producing them. This can lead to the evolution of lower virulence if antitoxin efficacy is high (Gandon *et al.* 2002; Vale *et al.* 2014).

Experimental demonstrations of vaccine-driven virulence evolution are limited but compelling, especially when combined with evidence from observational studies. The first demonstration was in the mouse-malaria system (Mackinnon *et al.* 2008). Replicate lines of malaria parasites were passaged through either naive or immunized mice for 18 passages; although virulence increased in both lines (as has been repeatedly demonstrated by serial passage experiments that reduce the transmission constraint on high virulence Ebert (1998)), virulence was significantly higher in lines passaged through vaccinated mice (Mackinnon and Read (2004); see also Barclay *et al.* (2012)). Vaccines have driven the evolution of *Bordetella pertussi* (the agent of whooping cough) (Octavia *et al.* 2011), and more virulent strains have been associated with the reemergence of pertussis in the developed world (Mooi *et al.* 2009). Previous empirical and theoretical work suggests that these changes in virulence factor expression were likely in response to vaccines which target toxin production (Mooi *et al.* 2001; van Boven *et al.* 2005). Marek's disease virus (MDV), a disease of poultry, has also evolved towards greater virulence since the introduction of vaccination in the 1950s (Witter, 1997; Atkins *et al.* 2013). These vaccines enhance survivorship, but do not prevent infection, slow within-host growth, or prevent transmission; the theory cited above would therefore predict they would select for increased virulence. Indeed, recent empirical evidence suggests that vaccinated chickens become transmission reservoirs for high-virulence MDV, leading to the persistence of strains that would otherwise ‘burn out’ (Read *et al.* 2015). On the other hand, antiretroviral therapy in HIV, which blocks transmission, has been suggested as a contributing factor to the decline in HIV virulence over time (Payne *et al.* 2014). More generally, evidence for vaccine-induced disease evolution is mounting for many diseases (Gandon and Day, 2008; Mackinnon *et al.* 2008), but for most of these we do not know yet how parasite life history is evolving.

## MULTIPLE INFECTION AND THE EVOLUTION OF VIRULENCE

Multiple infection can arise because a host is infected with multiple strains of the same parasite or with multiple species of parasite. Infection with multiple strains of the same parasite can result from separate infection events of co-circulating strains, or because the parasite has a rapid mutation rate and generates strain diversity through mutation within the host. Biologically, distinctions between ‘types’ of multiple infection matter because they affect the relatedness of the co-infecting parasites and relatedness can influence the optimal virulence strategy for the parasite. For clarity and simplicity, we will use the term ‘strains’ to refer to the different parasites infecting a host, acknowledging that different ‘strains’ may actually represent different species of parasite.

There have been three primary theoretical frameworks used to study multiple infection. Under a superinfection framework, each host is infected by a single strain at a time, but strains can displace one another based on strain traits, like virulence (Levin and Pimentel, 1981). Under a coinfection framework, each host is capable of being infected by one or more (typically two) strains simultaneously. Mathematically, the difference between these two frameworks is that in superinfection models, within-host competition is infinitely fast, whereas in coinfection models, within-host competition occurs explicitly but replacement of one strain by another is generally infinitely slow (Alizon and van Baalen, 2008). Coinfection models tend to be more analytically intractable and can bias evolutionary predictions, if care is not taken in model formulation (Lipsitch *et al.* 2009; Alizon, 2013*a*). Under a kin selection framework, the relatedness of strains and the fact that overall virulence may depend on the collective action of co-infecting strains is taken into consideration (Frank, 1992; Brown, 2001). However, kin selection studies typically lack epidemiological feedbacks, developing predictions from a simple fitness expression for the parasite (Alizon and Michalakis, 2015); incorporating such feedbacks can alter the predictions in important ways (Alizon and Lion, 2011).

Theoretical and empirical studies can be profitably summarized by considering the mode of interaction among co-infecting parasites. Parasites may compete or cooperate with one another. Competition may be mediated by resources, by the immune response, or by direct interference (Mideo, 2009; Read and Taylor, 2001). Cooperation may be mediated by collective action or public goods production (Brown, 2001; West and Buckling, 2003).

## COMPETITION FOR RESOURCES

Superinfection is an implicit model of resource competition, assuming that faster replicating, more virulent strains immediately displace one another within single hosts. As one might expect, this drives the evolution of higher virulence (Levin and Pimentel, 1981; Nowak and May, 1994). Additionally, superinfection modifies the simple predictions of trade-off theory. For example, if superinfection is possible, increasing the host background mortality rate will reduce the force of infection (by lowering the number of infected hosts); this reduces superinfection, thereby lowering virulence (Nowak and May, 1994; Gandon *et al.* 2001*a*; Bolzoni and De Leo, 2013; van Baalen and Sabelis, 1995*a*). By a similar line of reasoning, vaccination that blocks infection or transmission will lower the force of infection and reduce virulence (Gandon *et al.* 2001*b*; Gandon and Day, 2007).

In models where hosts can be infected by more than one strain at a time, the effect of resource competition on virulence evolution depends on the relatedness of strains and on how the overall virulence of a multiple-infection depends on the virulence of each infecting strain (Alizon, 2008*a*). If strains are unrelated and virulence increases competitive ability, then increasing competition will lead to the evolution of increased virulence (Bremermann and Pickering, 1983; May and Nowak, 1995; van Baalen and Sabelis, 1995*a*; Mosquera and Adler, 1998; Choisy and de Roode, 2010) unless overall virulence is negatively related to the distance between strains (Alizon, 2008*a*). On the other hand, if strains are closely related, then virulence is expected to evolve to lower levels as there is an inclusive fitness incentive to prudent exploitation (Frank, 1992, 1996).

Evidence for these predictions comes from a number of systems. In rodent malaria, increased virulence (as measured by red blood cell destruction, i.e. anemia) was positively associated with both within-host competitive ability and transmission success, such that the most virulent strain dominated in mixed-strain infections (de Roode *et al.* 2005; Bell *et al.* 2006). Similarly, in a *Daphnia*-bacteria host-parasite system, the most virulent (as defined by time to host death) strains dominated transmission, and virulence experienced by the host was approximately equal to the virulence induced by the most virulent strain (Ben-Ami *et al.* 2008; Ben-Ami and Routtu, 2013).

## APPARENT COMPETITION

Parasite strains and species may also interact through the immune response, if the presence of one parasite strain induces an immune response that is cross-reactive against other parasite strains or species (for example, through the stimulation of non-specific immune mechanisms or because of weak cross-immunity between closely-related strains) (Cox, 2001). This type of competition was first described in predator-prey models, and coined ‘apparent competition,’ as the increase in density of one species causes a decline in a second species, not because the two share a resource, but because they share a predator (Holt, 1977; Fenton and Perkins, 2010). Immune-mediated competition between parasites has been suggested to be the most important mechanism of within-host interaction, acting to inhibit (or promote) the coexistence of antigenically similar (or dissimilar) parasites (Read and Taylor, 2001). A particularly extreme example is the concomitant immunity observed in helminth infections, wherein adult worms elicit an immune response that has no effect on themselves, but prevents the establishment of larval stages (Smithers and Terry, 1969; Brown and Grenfell, 2001). Whereas a considerable amount of theory has focused on the role of immune-mediated apparent competition in maintaining parasite diversity, especially in antigenically diverse diseases like influenza (e.g. Ferguson *et al.* 2003), very little has been done to study how cross-immunity shapes virulence evolution (but see Best and Hoyle, 2013, e.g. where cross-immunity depends on virulence).

Alizon and van Baalen (2008) study virulence evolution using a model that considered partial cross-immunity between strains. Their model predicts that a high probability of coinfection leads to higher virulence. However, this analysis held the cross-reactivity of the immune system fixed, so it is unclear how weakening or strengthening this would affect virulence evolution. Moreover, the probability of coinfection was assumed independent of cross-reactivity, an assumption that is clearly violated in systems where parasites ‘vaccinate’ themselves against coinfection by inducing an immune response (Brown and Grenfell, 2001).

How immune-mediated apparent competition should affect virulence evolution is also unclear from an empirical perspective. Some early experiments with rodent malaria suggested that apparent competition might explain why avirulent strains suffer more in competition with virulent strains in immunocompetent mice than in immunocompromised mice (Raberg *et al.* 2006), but later work overturned that hypothesis (Barclay *et al.* 2008; Grech *et al.* 2008). Thus, while the importance of apparent competition for epidemiological dynamics is not in doubt, it is quite unclear how it affects virulence evolution.

## INTERFERENCE COMPETITION

Interference competition has only been theoretically studied from a kin selection perspective, which considers interference through spiteful competition. Spite, in an evolutionary context, is defined as an action that carries a direct cost to both actor and recipient; a classic example is the production of bacteriocins, antimicrobial compounds that are often highly specific and act to reduce competition by killing competitors, but are costly to the individual to produce (up to and including death of the producer Gardner *et al.* 2004). Simple models of spiteful competition among parasites assume that virulence is positively correlated with parasite growth rate, and find that virulence peaks at very low or very high relatedness (Gardner *et al.* 2004). This is because bacteriocin production peaks in infections with intermediate strain relatedness: at low relatedness, there are not enough closely related strains around to make the inclusive fitness benefit, outweigh the cost of production; at high relatedness, there are not enough distantly related strains around to make the benefit outweigh the cost.

There have also been strong empirical tests of this prediction. Massey *et al.* (2004) and Bashey *et al.* (2012) found that when parasites behave spitefully by producing bacteriocins, overall virulence, measured by host mortality, was lower in hosts with mixed-strain infections than in hosts with single-strain infections. Lower virulence (host mortality) of mixed-strain infections of intermediate relatedness was also found in (Inglis *et al.* 2009). An experimental evolution study with *Bacillus thuringiensis* showed that mixed-strain infections selected for spiteful interactions, and that mixed-strain infections were always less virulent (host mortality) than single-strain infections (Garbutt *et al.* 2011).

## PARASITE COOPERATION

If instead overall virulence depends on some form of collective action or public goods production, then the predictions reverse: virulence tends to go up with relatedness, rather than down (Brown, 2001; West and Buckling, 2003). Mechanisms that increase the intensity of within-host competition will tend to reduce virulence by reducing the relatedness of co-infecting strains (Brown, 2001; West and Buckling, 2003). However, this simple prediction comes from models ignoring epidemiological feedbacks, and more recent work has shown that the shape of any epidemiological trade-offs (e.g. the virulence-transmission trade-off); under some trade-off shapes, parasite cooperation may break down completely (Alizon and Lion, 2011).

A classic example of virulence dependent on collective action is found in siderophore-producing bacteria (West and Buckling, 2003). In this system, individual bacteria can produce iron-scavenging molecules called siderophores that bind host iron for bacterial uptake; importantly, these siderophores are a public good that can be utilized by any bacterium, regardless of whether they produce them or not. Overall virulence depends on the population growth rate of the parasite, which is dependent on siderophore production. Experiments in *Pseudomonas aeruginosa* have shown that siderophore production evolves when strains are closely related (Griffin *et al.* 2004). Further work has shown that infections with siderophore-producing (‘cooperating’) strains were more virulent (in terms of host mortality rate) than infections with mixes of cooperators and ‘cheaters’ that do not produce siderophores (Harrison *et al.* 2006). Moreover, cheaters attained higher densities in mixed-strain infections. This example provides the exception that proves the general rule that more virulent strains tend to be more competitive.

Similar results were found in studies of quorum sensing, another example of cooperative virulence characterized by the production of molecules that can be exploited by all individuals, regardless of whether they produce the molecule or not. Experiments in both *Pseudomonas* and *Staphylococcus aureus* have shown that high strain relatedness in infections leads to the competitive dominance of cooperators, and higher virulence to the host (measured by within-host growth rate or host mortality), whereas low relatedness leads to the persistence of cheater strains and lower virulence (Rumbaugh *et al.* 2012; Pollitt *et al.* 2014).

Another example comes from RNA viruses that produce shared intracellular products that aid in RNA replication (Turner and Chao, 1999). When multiplicity of infection was high (and many viral strains were competing within host cells), virulence evolved to a lower level as cheater strains that did not produce the intracellular products were able to invade. When single strains were competing within host cells, virulence remained high throughout the experiment. These experimental results provide robust support for the empirical predictions. Interestingly, and perhaps tellingly, the mechanistic basis for parasite virulence is known in all cases.

## INSIGHTS AND FUTURE PROGRESS

Virulence evolution is a compelling, and challenging, study subject because parasite evolution is shaped by the interaction of processes happening at two distinct scales: within-host and between-host. In order to persist, virulence evolves to balance the potentially conflicting selection pressures arising at these scales: rapid within-host growth may allow a parasite to outcompete co-infecting strains or species and to avoid clearance by the immune system, but at the cost of reduced host lifespan. Whether such within-host growth will be favored will depend on how well that parasite is able to spread to new hosts. Moreover, epidemiological dynamics may feed back on the within-host level: as the density of hosts infected with each strain changes, this alters which strains are likely competing within individual hosts (Coombs *et al.* 2007; Alizon, 2008*b*). The complexity of the problem has led to the development of an expansive theoretical literature studying how virulence evolves under different epidemiological, ecological and evolutionary settings. All of the theory we have reviewed here has dealt (implicitly or explicitly) with epidemiological processes, since ultimately, parasites must be successful at the population level to leave an evolutionary mark.

Our review suggests a key insight into virulence evolution: any mechanism that reduces the density of susceptible hosts will select for a decrease in transmission rate. If transmission and virulence are coupled via genetic constraints, as is assumed by most trade-off theory, then we would expect virulence to decrease as well. (Note that host mortality rate, which is assumed to apply equally to both uninfected and infected hosts, is not a mechanism that reduces susceptible host density; Day and Proulx (2004).) Spatial structure can decrease the density of susceptibles by reducing connectivity, leading to self-shading (Boots and Mealor, 2007). Vaccines that reduce the probability of becoming infected essentially reduce the density of susceptible individuals in the population (Gandon and Day, 2007). Density of susceptibles changes over an epidemic as a matter of course (Day and Proulx, 2004; Bolker *et al.* 2010). Exactly why reducing the number of susceptibles reduces virulence is multifaceted. At the between-host scale, reducing the number of susceptible hosts reduces the selective advantage of transmission, and if transmission is coupled to virulence then virulence will decrease as well (Day and Gandon, 2007). At the within-host scale, reducing the density of susceptible hosts reduces the force of infection, thereby reducing the intensity of within-host competition and the attendant advantage to virulent strains. Thus, reducing susceptible host density causes selection pressures arising at both the within-host and between-host scale to point in the same direction, which may explain why this prediction has not been contradicted by any empirical study of which we are aware (except, of course, in systems where virulence depends on collective action, e.g. Griffin *et al.* 2004).

Outside of identifying broad and robust theoretical predictions, the purpose of this review was to evaluate the empirical evidence for these predictions. Previous authors have noted that the theoretical and empirical literatures are not easy to reconcile (Ebert and Bull, 2003; Bull and Lauring, 2014). In part, this is because the theoretical quantities implicated as being critical to models of virulence evolution (like mortality rate and transmission rate) are notoriously difficult to measure (Alizon *et al.* 2009). Moreover, whereas most of the theory is focused at the scale of the population, most of the experiments are done at the scale of the individual. Because of these challenges, ‘tests’ of theoretical predictions are typically only approximate, using system-specific proxies for transmission and virulence. As we noted above, very few of the empirical studies we cite actually measured the relevant traits, a fact which must be taken into consideration when weighing the evidence for the theoretical predictions. Unfortunately, it is typically not known whether the evolutionary dynamics of such proxies will be the same as the evolutionary dynamics of the quantities studied by the theory – we have at least some evidence that, in fact, they will not (Day, 2002*a*).

What can be done to address this problem? We take heart from the number of very compelling studies, reviewed above, that brought theory motivated by a specific biological system together with experiments to study virulence evolution. For example, Berngruber *et al.* (2013); Berngruber *et al.* (2015) elaborated on the general mathematical theory to develop models of the *E. coli-λ* phage system. These models included system-specific epidemiological details like the possibility of the virus either lysing the cell (leading to horizontal transmission) or integrating into the cell's genome (leading to vertical transmission) and the potential for integration to prevent superinfection. They then used these models to predict key features of the evolutionary process and tested those predictions directly, a key advantage of working in bacteria-phage host-parasite systems. However, this approach has been successful in other systems as well: Gandon *et al.* (2001*a*) was able to explain the experimental results of Ebert and Mangin (1997) (discussed above in the section on host mortality) by constructing a mathematical model that mimicked the system and experimental design.

In both of those systems, it was possible to collect data and carry out experiments at both the within-host and between-host scales, which will not be true for most host-parasite systems. Two other approaches may be useful in these scenarios, both of which make use of the data that can be collected from within-host experiments.

Nested models (*sensu* Gilchrist and Sasaki, 2002), embed an explicit model for within-host dynamics into an epidemiological model. In essence, they allow epidemiological parameters (like those of the *R*_0_ expression) to be determined by the dynamics of the within-host model, which captures interactions between parasite strains, the immune system and parasite resources (Mideo *et al.* 2008). A strength of these approaches is that they do not need to specify, a priori, any particular trade-off between epidemiological parameters; instead such trade-offs can emerge out of the dynamics of the within-host model (Ganusov *et al.* 2002; Gilchrist and Sasaki, 2002; André *et al.* 2003; Alizon and van Baalen, 2005; Gilchrist and Coombs, 2006). They also allow an explicit consideration of how conflicting selection pressures arise and are resolved (Coombs *et al.* 2007; Alizon and van Baalen, 2008). The within-host model can potentially be parameterized using data collected for individual-level experiments, thereby allowing extrapolation to the between-host level on the basis of empirical data. There have been some interesting studies moving in this direction (e.g. Luciani and Alizon, 2009; Lythgoe *et al.* 2013), but none yet that has been directly parameterized on the basis of data. One potential downside to the nested model approach is that we often do not know enough about the interaction between the pathogen, within-host resources and the immune response to develop an appropriate within-host model. An approach has been developed to side-step this difficulty by working at a phenomenological level. This approach treats transmission, virulence and clearance as function-valued traits in a quantitative genetic setting (Day *et al.* 2011). Mideo *et al.* (2011) illustrate how this technique can be applied using data from malarial parasites but this approach has not yet been widely applied to determine how well it is able to predict evolutionary dynamics.

Another interesting potential avenue for future theory development and theory/data integration is motivated by a consideration of the relative sensitivity of parasite fitness (Frank and Schmid-Hempel, 2008). This verbal theory focuses on the fact that virulence arises out of particular pathogenic mechanisms, and those mechanisms have two adaptive functions for the parasite: they can act to enhance transmission or to evade or inhibit clearance by the immune system. The authors argue that parasite virulence will be driven by the evolution of mechanisms that reduce clearance, rather than mechanisms that enhance transmission, because parasite fitness will be more sensitive to changes in clearance-enhancing traits. This framework suggests an approach to identify key virulence-determining traits in natural host-parasite systems. For example, parasites differ greatly in the dose required to successfully establish an infection, which may in part be explained by the mechanism used to evade the immune response. If the mechanism acts locally (e.g. via molecules attached to the surface of the parasitic cell), infectious dose will be small; if the mechanism acts at distance (e.g. via diffusible molecules), infectious dose will be large (Schmid-Hempel and Frank, 2007). Moreover, these differences in mechanism may explain virulence differences: diffusible pathogenic molecules act over a much greater spatial extent and will build up over the course of an infection, leading to high virulence. A recent comparative study confirmed the former prediction, but not the latter: infectious dose was significantly higher in human pathogens with distantly acting molecules, but virulence was strongly negatively associated with infectious dose (Leggett *et al.* 2012). However, that the predictions based on the sensitivity framework were not borne out does not detract from its potential utility; indeed, the fact that the theory made a prediction that was testable from existing data should be seen as a strength of the approach.

Despite the considerable theoretical literature surrounding virulence evolution, there is still a clear need for general theory in some areas. At the most basic level, almost all of the work summarized here assumed the existence of a trade-off between virulence and transmission. Other potential trade-offs, such as between virulence and recovery (Anderson and May, 1982; Frank, 1996) or between transmission and recovery (Read and Keeling, 2006; Alizon, 2008*b*) have been critically understudied. Even within the context of transmission-virulence trade-off theory, important epidemiological and ecological processes have not been studied. In particular, very little is understood about how immune-mediated apparent competition might affect virulence evolution, despite its importance in nature (Cox, 2001; Read and Taylor, 2001). There is also a dearth of theory focused on how other ecological interactions, especially those of the host with its own resources, will interact with epidemiological processes to affect virulence evolution (Becker and Hall, 2014; Cressler *et al.* 2014).

However, given the challenge of experimentally measuring the quantities implicated by current theory as crucial in shaping virulence evolution, we feel that the onus is on theoreticians to better connect mathematical models with empirical data. This connection could come through developing models of specific systems or by developing general theory that better captures common empirical measures of virulence, such as host morbidity or physiological condition.
